# ID 79 - Lenalidomida em Combinação com Rituximabe para o Tratamento do Linfoma Folicular (LF) em Indivíduos Previamente Tratados: análises de custo-efetividade e de impacto orçamentário

**DOI:** 10.5327/2237-9622.2025.v34s1.79

**Published:** 2025-11-25

**Authors:** Maiara Silva Araujo, Ludmila Peres Gargano, Layssa Andrade Oliveira, Haliton Alves de Oliveira, Rosa Camila Lucchetta

**Keywords:** análise custo-benefício, avaliação da tecnologia biomédica, análise de sobrevida, linfoma folicular, técnicas de apoio para a decisão

## Abstract

**Introdução:**

O linfoma folicular (LF) é um tipo de linfoma não Hodgkin (LNH) caracterizado pela produção anômala de linfócitos B. No Brasil, excluindo os tumores de pele não melanoma, o LNH é o oitavo câncer mais frequente em homens e o nono em mulheres. O tratamento de primeira linha para o LF geralmente oferece uma resposta satisfatória e duradoura. Contudo, alguns pacientes podem experienciar recidiva ou progressão após um período variável de remissão. As opções de tratamento padrão para LF recidivado são limitadas. Atualmente, no Sistema Único de Saúde (SUS), está disponível para LF que progrediu ou recidivou após o tratamento inicial apenas a combinação de rituximabe (RTX) com quimioterapia. Entretanto, alternativas estão disponíveis no mercado, como a combinação de lenalidomida com RTX(LR), um agente imunomodulador que mostrou aumentar a sobrevida global (SG) e da sobrevida livre de progressão (SLP) em pacientes com LF. Desta forma, o objetivo desta análise foi avaliar a razão de custo-efetividade incremental (RCEI) e o impacto orçamentário da LR no tratamento de pacientes com LF previamente tratados, comparada ao RTX+quimioterapia, sob a perspectiva do SUS.

**Método:**

Foi conduzida uma análise de custo-utilidade para anos de vida ajustados pela qualidade (AVAQ), utilizando um modelo de sobrevida particionada (PartSA). As curvas de SG e SLP do grupo comparador extraídas do ensaio clínico AUGMENT para LR foram extrapoladas para um horizonte de 40 anos utilizando diferentes distribuições paramétricas. A seleção das curvas utilizadas no modelo foi baseada em testes de informações criteriais (AIC/BIC) e inspeção visual. As curvas foram ajustadas com os HR da comparação entre LR versus RTX. Valores de utilidade foram obtidos da literatura internacional e foram considerados apenas custos diretos médicos. Para a análise de impacto orçamentário (AIO), os pacientes elegíveis foram estimados a partir da demanda aferida, e assumiram-se dois cenários com diferentes market share, que foram comparados ao cenário referência (sem LR).

**Resultados:**

No caso-base, utilizou-se a distribuição exponencial para SG e SLP. A LR apresentou benefício incremental (3,10 AVAQ), com custos incrementais da ordem de R$ 187.192. A RCEI sugeriu que a LR pode ser custo-efetiva (RCEI de R$ 60.298 por AVAQ), considerando o limiar de R$ 120.000 por AVAQ ganho, adotado para doenças graves. Nas análises de sensibilidade, a lenalidomida manteve-se custo-efetiva na maioria dos cenários. No entanto, quando o HR da SG foi ajustado para o limite superior do intervalo de confiança (0,92), a RCEI ultrapassa o limiar de custo-efetividade proposto, refletindo a maior incerteza associada a este parâmetro no modelo. Na AIO, a incorporação da LR resultaria em um impacto orçamentário acumulado ao longo de cinco anos de R$ 55.625.601 se o market share atingisse 80% no quinto ano, e de R$ 30.557.046 se o market share alcançasse 50% no quinto ano.

**Conclusão:**

Indivíduos com LF previamente tratados podem se beneficiar da combinação LR, quando comparado ao tratamento padrão com RTX+quimioterapia. A RCEI mostrou que a lenalidomida pode ser custo-efetiva, com um RCEI de R$ 60.298 por AVAQ ganho.

